# Geophysical field zoning for nitrogen fertilization in durum wheat (*Triticum durum Desf*.)

**DOI:** 10.1371/journal.pone.0267219

**Published:** 2022-04-27

**Authors:** Michele Denora, Mariana Amato, Gennaro Brunetti, Francesco De Mastro, Michele Perniola

**Affiliations:** 1 Dipartimento delle Culture Europee e del Mediterraneo, Università degli Studi della Basilicata, Matera, Italy; 2 Scuola di Scienze Agrarie, Forestali, Alimentari ed Ambientali, Università degli Studi della Basilicata, Potenza, Italy; 3 Dipartimento di Scienze del Suolo, della Pianta e degli Alimenti, Università di Bari, Bari, Italy; Ghazi University, PAKISTAN

## Abstract

The current social context requires an increase in food production, improvement of its quality characteristics and greater environmental sustainability in the management of agricultural systems. Technological innovation plays a great role in making agriculture more efficient and sustainable. One of the main aims of precision farming (PF) is optimizing yield and its quality, while minimizing environmental impacts and improving the efficient use of resources. Variable rate techniques (VRT) are amongst the main management options for PF, and they require spatial information. This work incorporates maps of soil properties from low induction electromagnetic measurements into nitrogen (N) balance calculations for a field application of VRT nitrogen fertilization of (*Triticum durum* Desf., var. Tirex). The trial was conducted in 2018–19 at Genzano di Lucania (PZ, Italy) geologically located on the clayey hillsides of the Bradanica pit and the Sant’Arcangelo basin. Three soil homogeneous areas were detected through low induction electromagnetic measurements and used as uniform management zones. The amount of nitrogen fertilizer to be applied by VRT was calculated on the base of estimated crop nitrogen uptake and soil characteristics of each homogeneous area. Crop response to VRT was compared to uniform nitrogen application (UA) on the whole field. The application of VRT resulted in a reduction of 25% nitrogen fertilizer with the same level of yield respect to UA. Grain protein content, as well as gluten content and N content, were significantly higher in VRT than in UA. As a consequence of lower nitrogen input and higher levels of N removal, VRT reached a higher nitrogen use efficiency than UA, and this indicates a lower environmental impact and a higher economic profitability.

## 1. Introduction

Effective nutrient management is key for future challenges linked to sustainable development. Specific issues include avoiding environmental losses and preserving or improving yield and quality of crops [1, 2].

Nitrogen is one of the main constraints limiting crop yield [3], especially for cereals where low nitrogen or poor nitrogen management reduce yield, residual soil fertility, quality and environmental sustainability [4, 5]. However, the massive use of N fertilizers is harmful for terrestrial and water ecosystems, air pollution as well as human health [6], with only 33% actually used by plants [7]. Nitrogen use efficiency (NUE) is the fraction of applied nitrogen that is taken up and used by crops, therefore increasing NUE by optimizing times and rates of application is crucial for improving sustainable and productive agriculture [4].

Optimizing N may be pursued through timing and choosing stabilised forms of N [8] or through spatial management [9, 10], or precision dosing based on crop vegetation indices [11]. Different level of soil N mineralization, leaching, volatilization and crop uptake, generate a spatial variability of N in the soil, justifying the application of variable rate techniques (VRT) to N fertilization [12]. Durum wheat is a staple in the Mediterranean area where traditional uniform within-field management results in low time and space efficiency [13]. Precision farming (PF) supported by the use of different technologies [14–16] is an important approach to address nitrogen efficiency through differential management in space.

Criteria for fertilizing different zones in the same field vary, and the debate as to whether lower-yielding field regions should be fertilized with lower or higher rates of N is still under research [17] and the analysis implies agronomic and economic issues [18].

A classical problem in PF is the definition of uniform management zones, i.e., field regions within which agronomic practices should be applied uniformly, and different enough from other regions that management should differ between them. Earlier approaches were based on the analysis of time-series of data on crop behaviour, such as multi-year yield maps or vegetation indices [9]. Geophysical mapping of soil properties has proved capable of detecting soil features related to crop behaviour [11, 19] and more specifically to wheat yield and quality [20], and has thereafter been used to delineate uniform management zones [21]. Criteria for incorporating geophysical mapping in PF management range from using geophysical data alone [22], to coupling them with other properties of fields and crops. The coupling of soil data and crop yield was used by Guerrero et al. [17] to compare N fertilization strategies in barley and wheat. The joint analysis of geophysical soil maps and plant properties implies the need of addressing complex features of different spatial datasets with measures that are repeated in time [23, 24]. Overall, field zoning for PF is based on spatial data, but new principles of PF need to be combined with well-defined principles of plant nutrition, soil chemistry and chemistry of the fertilizer elements. Nitrogen balance is a classical agronomic fertilization criterion based on predictions of nitrogen uptake considering the actually obtainable yield; corrections are then applied for soil nitrogen content and interactions between the fertilizer and the main physical and chemical soil parameters [25].

This work aims to use geophysical soil mapping coupled with differential nitrogen balance as a basis for the rapid delimitation of uniform management zones for the application of variable rate nitrogen fertilization in durum wheat.

## 2. Materials and methods

### 2.1 Field trials

**Fig 1:**
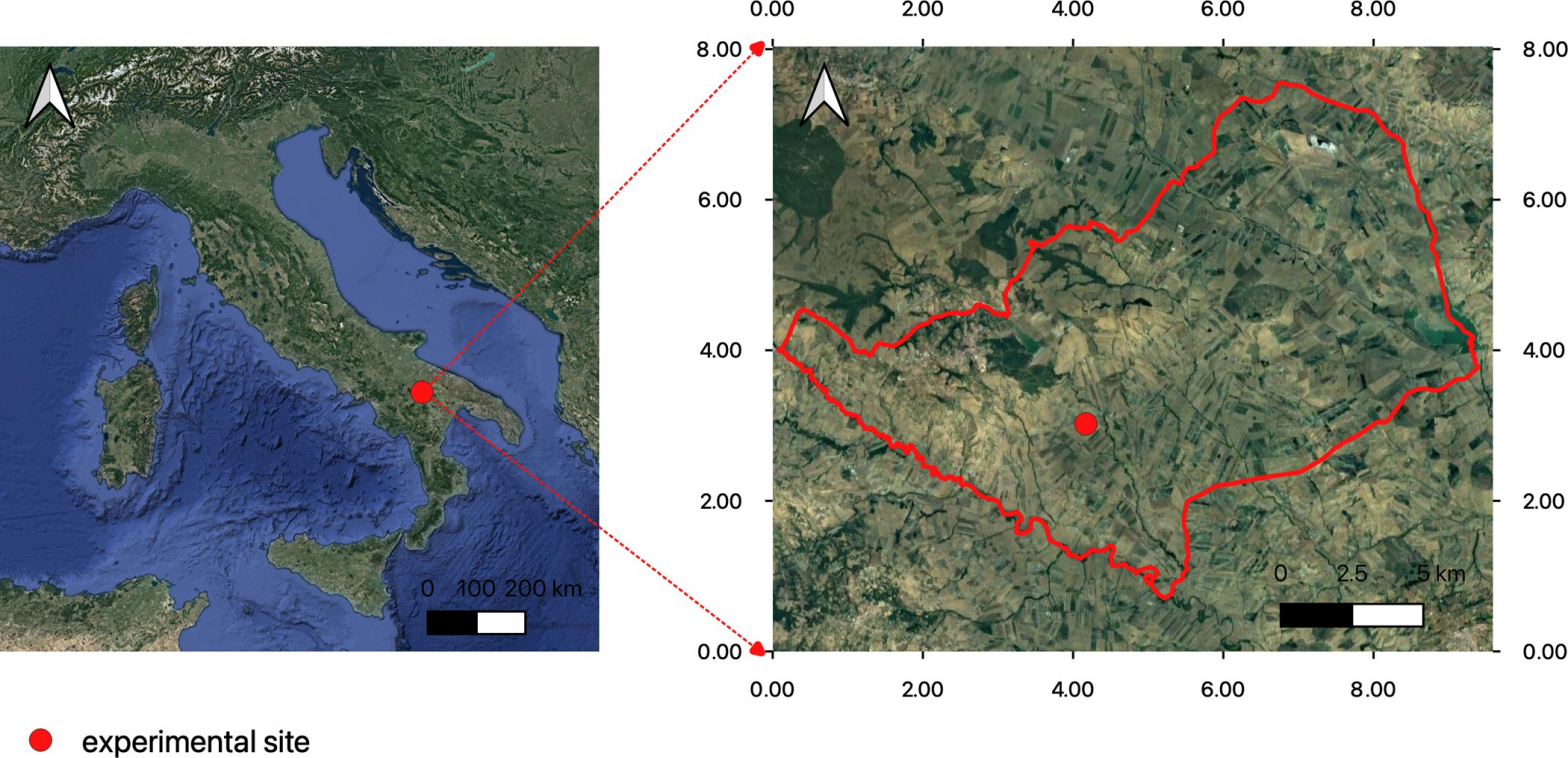
Location of experimental site.

The trial was conducted in 2018–19 at Genzano di Lucania (PZ) latitude: 40.82° N, longitude: 16.08° N. The study area (4,07 ha^-1^) is located on the clayey hills of the Bradanica grave and the basin of Sant’Arcangelo (Fig 1).

**Fig 2:**
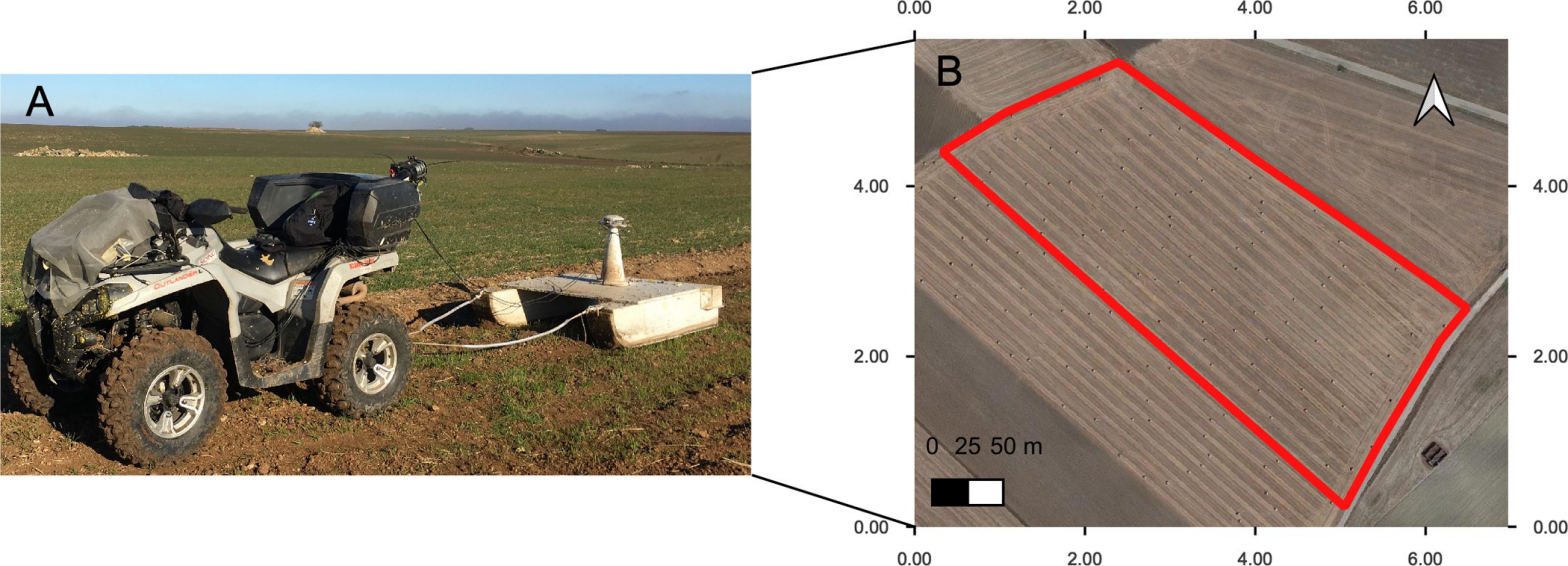
Soil spatial variability with electromagnetic induction technique. Low induction electromagnetic soil mapping (A, B), miniexplorer, GF Instruments (A), area tested by the instrument (B).

**Fig 3:**
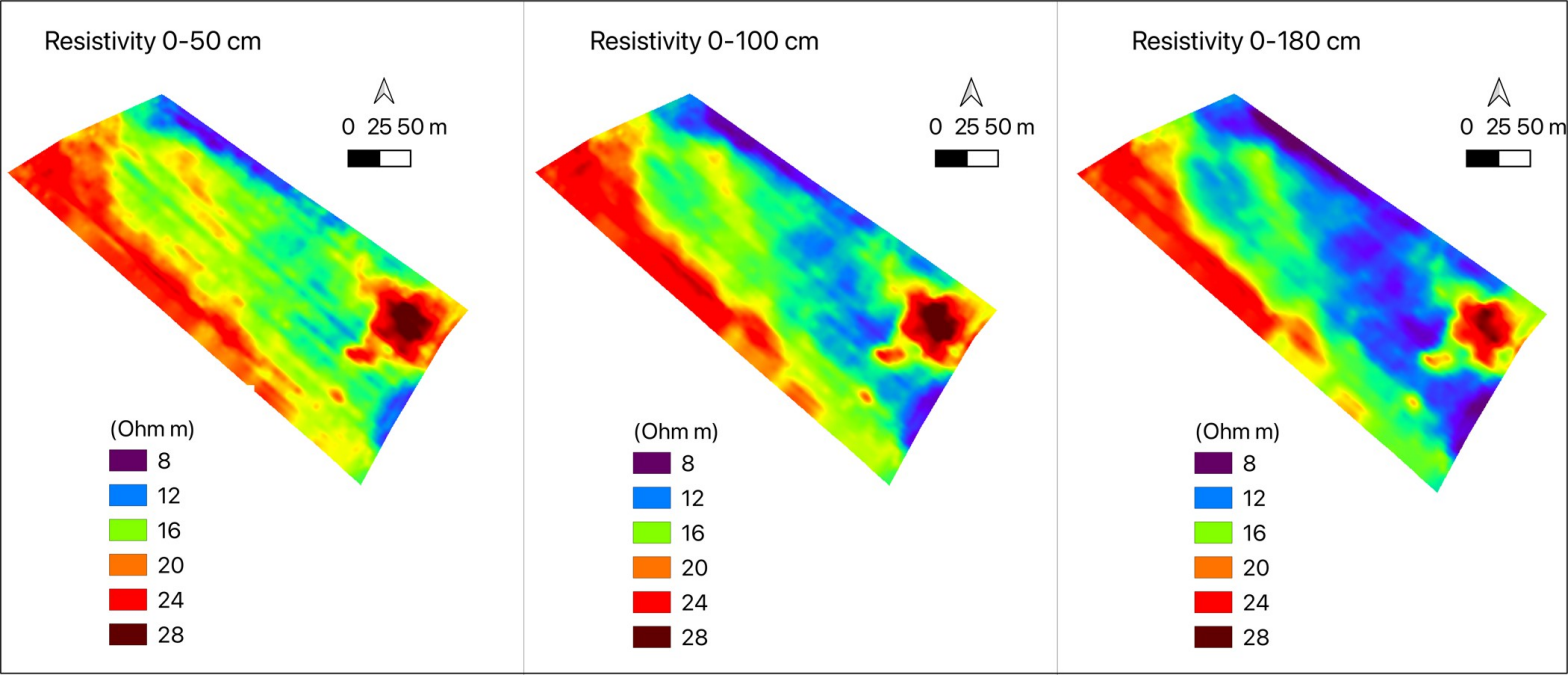
Resistivity maps of experimental field.

The soil spatial variability was detected by mean of low induction electromagnetic technique with a Miniexplorer (GF Instruments Brno-CZ) (Fig 2A) detecting bulk soil electrical conductivity (Cb) at three depths (0–50 cm, 0–100 cm and 0–180 cm-. The survey was conducted with a distance of 6 m between transects and average measurement distance of 0.8 m along transects (Fig 2B) and Cb values were converted to Electrical resitivity Rho = 1/Cb (Ohm m). Three electrical resistivity maps were obtained (Fig 3) [17, 18], and values of resistivity were averaged over the three depths. The field was divided in three zones according to the following average values: zone 1: 23.03 Ohm m, zone 2: 12.81 Ohm m and zone 3: 18.29 Ohm m. The coefficient of variation of resistivity values within each zone was: zone 1: 13%, zone 2: 12%, zone 3: 11%. The surface areas of the three zones were: area 1: 0.29 ha^-1^; area 2: 1.9 ha^-1^; area 3: 1.88 ha^-1^.

Across the whole field durum wheat (*Triticum durum L*., var. Tirex) was sown with inter row spacing of 0,13 m and 250 kg ha^-1^ of seeds were used. Soil tillage consisted in a 40 cm deep plowing (August 28 2018) and two harrowing (November 11 2018 and December 5 2018). A pre-sowing fertilization was broadcast applied with 92 kg ha^-1^ of P_2_O_5_ and 36 kg ha^-1^ of N.

We tested the hypothesis that variable-rate fertilization is more efficient than uniform application by comparing two N fertilization strategies: variable rate (VRT) and uniform (UA) on three replications in each of the areas identified through soil mapping. The amount of N fertilizer to be applied was calculated based on estimated crop N uptake and soil characteristics of each homogeneous area as follows: crop potential N uptake was estimated based on the crop yield of previous year in each homogeneous area, and was corrected considering the N contribution provided to the crop by the mineralization of the organic matter. N mineralization was calculated considering the content of organic matter in the soil profile explored by the roots, its content in organic N and by the mineralization efficiency which in turn depends on the carbon/nitrogen ratio of the soil (1 for C/N < 9; 0,5 for C/N >9, C/N<12). For the VRT treatment, the N doses applied in each area through a variable rate spreader are reported in Table 1. A dose of 35 kg ha^-1^ of N (UREA 46%) was spread in pre-sowing over the entire field. At the phenological stage of end tillering, a different rate according to the soil spatial variability was spread in each area at the end of tillering (Table 1). For each treatment we established plots of 2x2 m^2^ replicated three times inside each of the homogeneous areas identified in the field. In all such plots we applied a dose of N equal to 85 kg ha^-1^ (UA), which corresponds to the amount generally applied by the farmer, and slightly over the average of the dose of N applied in the three zones. The fertilizer was manually spread in UA.

**Table 1:** Units of N supplied in the different experimental zones.

Distribution mode	Zone	Dose of N (kg ha^-1^)	Dose of N (kg ha^-1^)	N_tot_ (kg ha^-1^)
		pre-sowing	end tillering	
**Uniform (UA)**	1	35	85	120
	2			
	3			
**Variable Rate (VRT)**	1	35	121	156
	2	35	63	98
	3	35	36	71

### 2.2 Soil analysis

Following the soil spatial variability determined by electrical resistivity, in each of the identified homogeneous areas, soil samples were collected at regular grid intervals in triplicate after the harvest at the depths of 0–40 cm and characterized by conventional analytical methods according to Page et al. [26]. All samples were air-dried and 2-mm sieved before laboratory analyses. Particle size distribution was determined by the pipette method after removing carbonates and organic matter and the textural class of the soil was identified by the USDA soil textural classification system [27]. The organic carbon (OC) content was measured by the Walkley‐Black method, and the total Kjeldahl N was determined by the Kjeldahl method. The available phosphorus (P_ava_) was determined by ultraviolet and visible (UV–vis) spectrophotometry according to Olsen method [26]. The total content of CaCO_3_ was determined by the gas-volumetric methods (Dietrich–Freuling calcimeter method), whereas the active lime was extracted with 0.1 M ammonium oxalate and determined by titration with 0.1 M KMnO_4_.

In Table 2, the main physico-chemical characteristics for the computation of N fertilization are reported.

**Table 2:** Main soil physico-chemical characteristics of the three experimental zones.

Soil Proprieties	Zone 1	Zone 2	Zone 3
**Sand 2.0–0.05 mm (%)**	56	27	32
**Silt 0.05–0.002 mm (%)**	25	32	31
**Clay < 0.002 mm (%)**	19	41	37
**Soil texture (USDA)**	Sandy Loam	Clay	Clay Loam
**Total N (g/kg)**	0.4	1.1	1.2
**Available phosphorus (P mg/kg)**	6.0	7.0	10.0
**Exchangeable potassium (meq/100g)**	0.3	1.1	1.0
**Organic matter (%)**	0.5	1.9	2.2
**Organic carbon (%)**	0.3	1.3	1.1
**C/N ratio**	7.9	9.4	10.2
**Cation Exchange Capacity (meq/100g)**	14.6	24.6	24.7
**pH**	8.3	8.2	8.1
**Aptitude for wheat cultivation**	marginal	sub-optimal	sub-optimal

### 2.3 Biomass yield and grain quality

At the harvest, plant samples on 1 linear meter were taken. Dry biomass weights were determined by drying samples at 70°C to constant weight. At harvest, was measured yield and its components (n ears/m^2^, n seeds/ear, total yield) and grain qualitative parameters (protein content, specific weight, gluten and yellow index with a FOSS Infratec 1241). On grain and straw, the N content was measured using a TOC analyzer (soli TOC® cube, Elementar, Hanau, Germany). Nitrogen use efficiency (NUE) was calculated as the ratio between total N uptake (calculated by multiplying the N concentration for dry biomass) by the crop of each experimental treatment and N applied with fertilizer [28].

### 2.4 Statistical analysis

The dataset was analyzed using “the lme4 package” of the “R” statistical software, version 3.6.3 [29]. After testing the basic assumptions of analysis of variance (ANOVA) as the normal distribution of the experimental error by Shapiro-Wilk’s test together and the homoscedasticity by means of the Levene test, the ANOVA model was performed. A split–plot design with three replicates considering zone, N fertilization (UA, VRT), zone x N fertilization has been used. All the factors were considered as fixed, while the replicates as random. The statistical significance of the difference among the means was determined using Tukey’s honest significance difference post hoc test at the 5% probability level.

## 3. Results

### 3.1 Weather conditions

**Fig 4:**
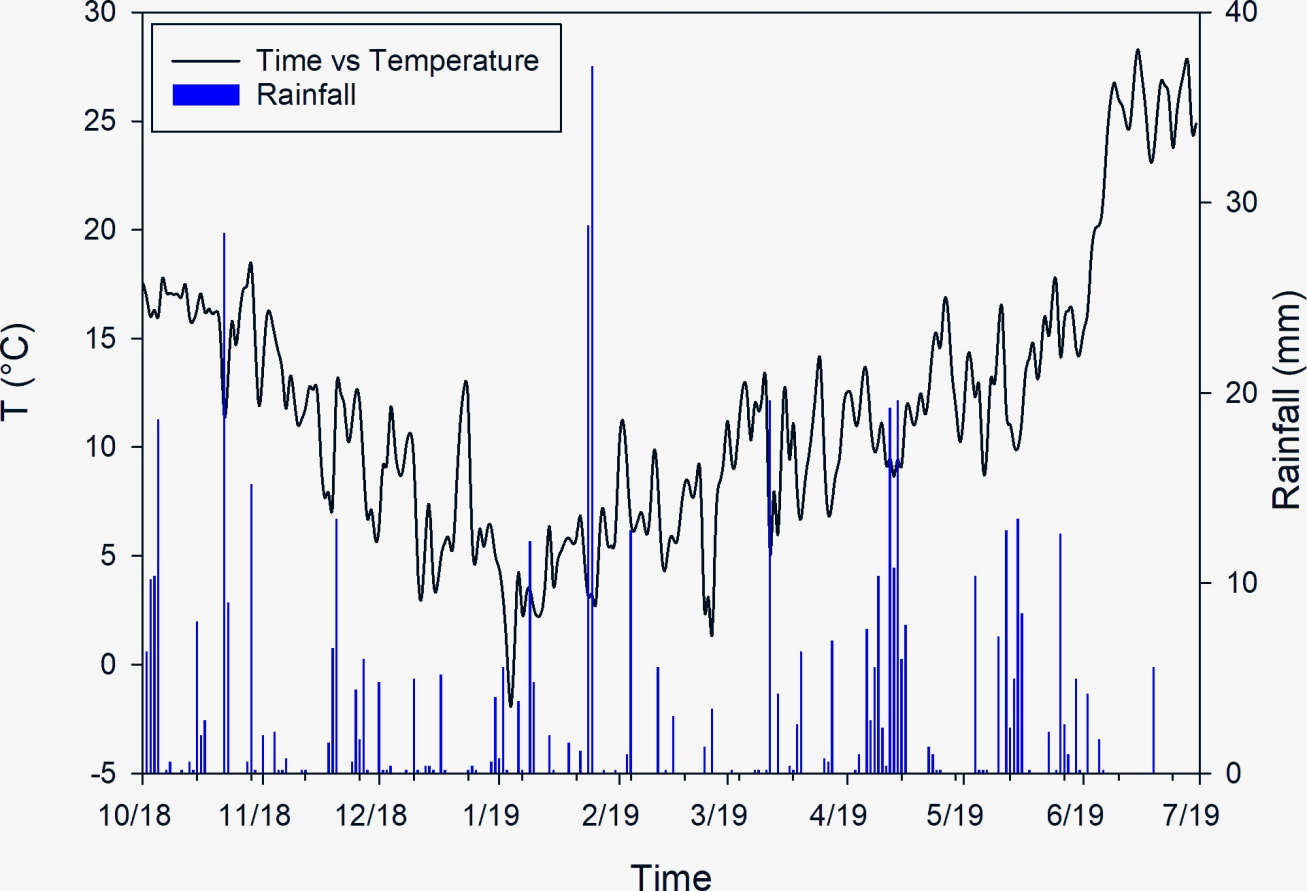
Weather conditions. Monthly average precipitation and temperature during the study period of winter wheat growing cycle (October 2018 to June 2019).

Temperature and precipitation recorded during the wheat growing period (October 2018 to June 2019) are reported in Fig 4. Heavy rainfall occurred in the period October-December 2018 (171.20 mm from 1/10 to 17/12), and this caused sowing on wet soil. Soil compaction due to wet conditions, resulted in a reduced emergence, and therefore a low density of plants and ears, particularly on the clay soil of zone 2.

January to February, were characterized by high temperature, with maximum temperatures reaching 20°C (Fig 4). From mid-April, the rains were prolonged and of strong intensity, exposing the crop to potential damage during the delicate phase of earing. The end of the vegetative cycle took place with high temperatures and almost total absence of precipitation.

### 3.2 Yield response

The total biomass and grain yield of zone 2 was significantly lower respect to that of both zones 1 and 3 (Table 3). Regarding straw yield, differences were significant between zones 2 and 3 only, whereas the number of ears m^-2^ was significantly different between all zones.

**Fig 5:**
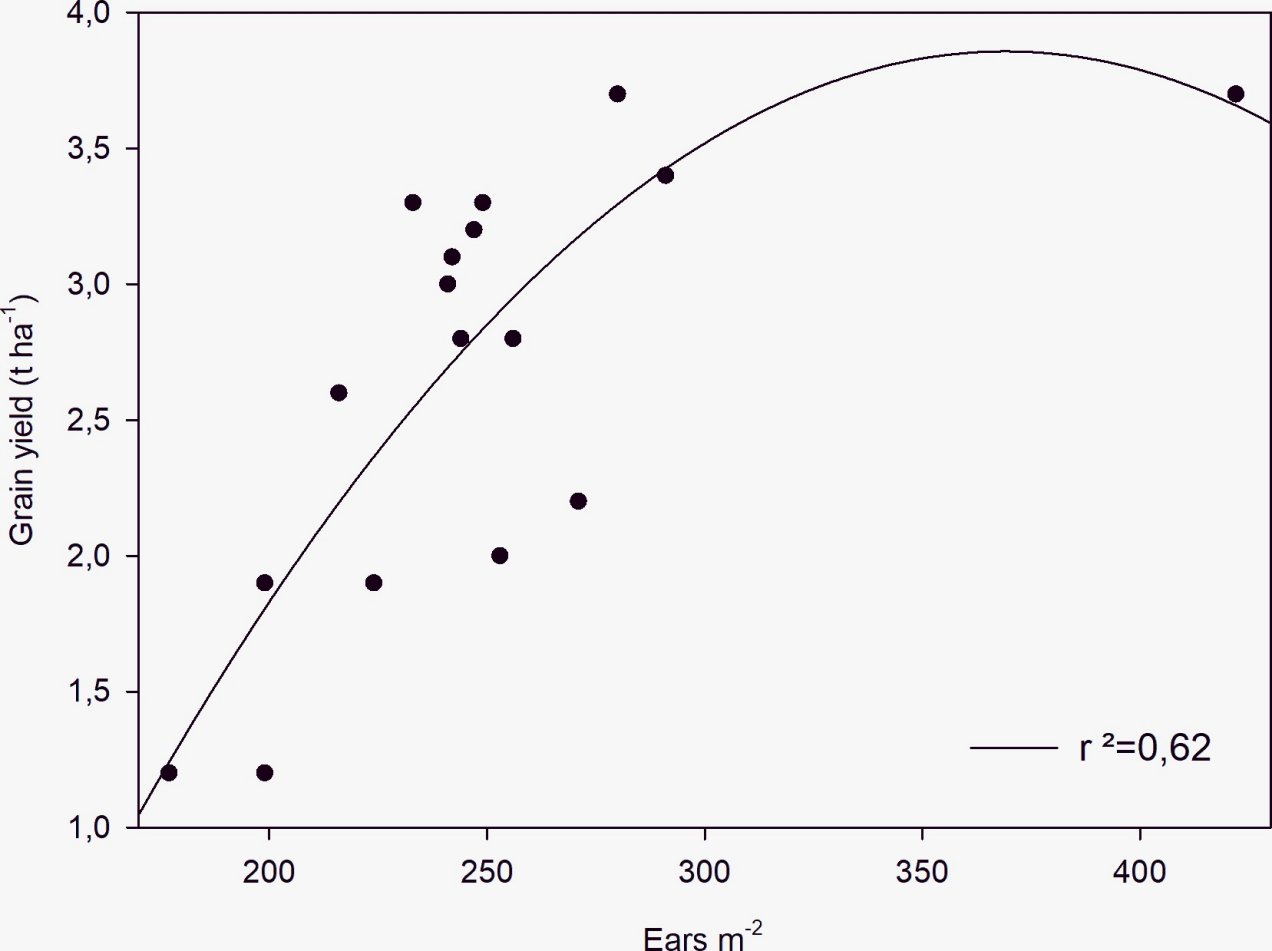
Relationship between yield and number of ears measured in the experimental conditions.

A significant relationship was found between the density of ears and grain yield across zones (Fig 5). No significant differences were detected between UA and VRT for yield parameters, in spite of different amounts of N applied with the two treatments.

**Table 3:** Biomass and yield in the different experimental conditions of durum wheat.

Treatment	Total biomass (t ha^-1^)	Grain yield (t ha^-1^)	Straw yield (t ha^-1^)	ears m^-2^
**Zone 1**	9.2 a	3.0 a	6.1 ab	247 b
**Zone 2**	6.5 b	1.7 b	4.8 b	221 c
**Zone 3**	10.8 a	3.2 a	7.5 a	282 a
***Significance***	***	***	**	*
**Zone 1 x UA**	9.1	3.1	6.1	244 ab
**Zone 1 x VRT**	9.2	2.9	6.2	251 ab
**Zone 2 x UA**	6.3	1.7	4.6	200 c
**Zone 2 x VRT**	6.7	1.8	4.9	241 ab
**Zone 3 x UA**	10.7	3.2	7.4	308 a
**Zone 3 x VRT**	10.9	3.2	7.6	256 ab
***Significance***	n.s	n.s.	n.s	*

Signif. codes: 0 ‘***’ 0.001 ‘**’ 0.01 ‘*’ 0.05 ‘.’ Significance at P<0.05; **, significance at P< 0.01; ns, no significant difference. Different letters indicate significant different.

Also, interaction of zone and N application method were not significant except for density of ears in zone 2 only, where differential application of N resulted in a significantly higher number of ears m-^2^ (Table 3).

### 3.3 Grain quality and nitrogen use efficiency

The qualitative grain traits measured at harvest are reported in Table 4. Zone 1 showed a significantly higher content of grain protein, yellow colour, gluten, and N compared to both zones 2 and 3. N uptake calculated on the basis of total straw + grain removal was lowest in Zone 2, whereas NUE showed a significant interaction between zone and distribution mode, since only in zone 3 the VRT is more efficient than UA. (1.68 vs 0.92). On the contrary, in positions 1 and 2 no significant differences were observed between UA and VRT in terms of NUE. Nevertheless, VRT in zone 3 is more efficient than all other treatments and UA in zone 1 and 3 is also more efficient than UA in zone 2.

**Table 4:** Qualitative traits and nitrogen use efficiency of durum wheat.

Treatment	Grain Protein (%)	Grain Yellow Color	Grain Gluten (%)	Grain N (%)	Tot. N Uptake (kg ha^-1^)	NUE tot.
**Zone 1**	15. 7 a	14.9 a	11.7 a	2.7 a	112.0 a	0.82 b
**Zone 2**	13.3 b	14.5 b	10.2 b	2.4 b	63.6 b	0.6 b
**Zone 3**	13.9 b	14.5 b	9.7 b	2.3 b	114.9 a	1.3 a
***Significance***	**	**	**	**	***	**
**UA**	13.7 b	14.6	10.1 b	2.4	96.5	0.78 b
**VRT**	14.9 a	14.6	11.1 a	2.5	97.1	1.03 a
***Significance***	**	ns	**	ns	ns	**
**Zone 1 x UA**	15.0	14.9	11.1	2.6	110.2	0.92 b
**Zone 1 x VRT**	16.4	14.9	12.4	2.8	113.8	0.73 bc
**Zone 2 x UA**	12.9	14.5	9.4	2.22	60.1	0.5 c
**Zone 2 x VRT**	13.6	14.5	10.0	2.34	67.0	0.68 bc
**Zone 3 x UA**	13.3	14.5	9.7	2.28	119.3	0.92 b
**Zone 3 x VRT**	14.6	14.4	10.8	2.50	110.6	1.68 a
***Significance***	ns	ns	ns	ns	ns	***

Signif. codes: 0 ‘***’ 0.001 ‘**’ 0.01 ‘*’ 0.05 ‘.’ Significance at P<0.05; **, significance at P< 0.01; ns, no significant difference. Different letters indicate significant different.

Grain protein and gluten were significantly higher in VRT, whereas the superiority of VRT in grain nitrogen percent and total uptake was not statistically significant. The value of nitrogen use efficiency in VRT across field zones was equal to 1.03, significantly higher than that of 0.78 in UA.

## 4. Discussion

Data from this experiment show that areas chosen on the basis of soil resistivity mapping within a field corresponded to different soil textures and crop behavior. In particular zone 2, characterized by clayey texture, showed lowest biomass, yield and nitrogen uptake, while zone the sandy-loam area (zone 1) had highest grain quality. Overall nitrogen use efficiency was highest in zone 3, a clay loam area within the field. The low yield in area 2 may be ascribed to the high content of clay and the consequent risk of waterlogging and poor tolerance of machine traffic in case of high precipitation during crucial times such as sowing. This interpretation is supported by the lower number of ears reported in Table 3 for this field region. Such constraints due to soil physical properties were therefore more effective in determining yield than the more favourable soil chemical characteristics of shown in Table 2. This is confirmed by the significant relationship we found between the density of ears and grain yield across zones in our field (Fig 5). This also means, that the computation of N supply based only on the soil characteristics is not sufficient. For an accurate computation of N supply by VRT techniques, the real crop conditions at the moment of fertilizations have to be considered. In accordance with Song et al. [30] soil characteristics should be combined with crop remote sensing for a more accurate computation of site-specific N fertilization.

In our data no significant differences were detected between UA and VRT for yield parameters, in spite of different amounts of N applied with the two treatments. This result could be ascribed to no changes in soil chemical parameters after the use of different doses of N fertilization [31, 32]. Also, interaction of zone and N application method were not significant except for density of ears in zone 2 only, where differential application of N resulted in a significantly higher number of ears m^2^ (Table 3). Therefore, N was able to compensate for the negative effects of a high clay content on plant density on the production of ears, which was correlated with yield.

Overall, results can be commented by saying that soil conditions dominated over N application in determining yield, and therefore the choice of N treatments needs to be made exclusively in terms of savings in N fertilizer, rather than in potential increases in yield. In the whole 4.07 ha^-1^ field the application of precision farming, gave as a result a reduction of 25% of N application in VRT respect to UA (373 Kg in VRT and 498 in UA Kg of N in 4,07 ha^-1^).

Although our data come from one year only, they confirm that if VRT is applied to N fertilization, the computation of N fertilizer rate considering only physico-chemical characteristics of the soil is not sufficient. Rossi et al. [19] found that the relationships between soil electrical resistivity and soil texture were linear while their effect on crop behaviour was strong but non-linear, therefore soil and crop data were both necessary for the correct identification of management zones. A sophisticated “informed clustering” [23, 24] approach identifies management zones within a field based on a function fitted on crop response versus geophysical mapping; this way different zones correspond to a different type and extent of influence of soil properties on plant behaviour. Our simplified approach based on geophysical mapping has the drawback of not allowing such discrimination but it is fast and can be applied before vegetation data is available.

Based on our data, the rate of N should be corrected considering also the actual plants density or, more in general, on the basis of the actual status of the crop measured at the time of fertilization, by mean of indices of crop density/status such as NDVI, as already suggested by Benincasa et al. [33].

In our experiment zone 1 showed a significantly higher content of grain protein, yellow colour, gluten, and N compared to both zones 2 and 3. As suggested by Diacono et al. [34], if this behaviour should be confirmed in subsequent years, the delineation of homogeneous areas taking quality into account could allow to segregate the harvested grain into different lots of semolina qualitative parameters. In all treatments, protein content was higher than 13.0%, therefore above the limit of 12.5% prescribed by the Italian law for a grain of good technological quality.

Regarding nitrogen uptake VRT showed high levels of N removal by the crop and a good protein content and other quality parameters in spite of lower N inputs, and this translated into a high nitrogen use efficiency. More specifically in VRT a large amount of the N given with the fertilizer was taken up by the crop, while in the case of UA 22% of the N applied was not, and therefore was left in the soil, and prone to leaching and pollution of deep soil/water. This has consequences on farm profitability but also on reducing environmental impact with respect to UA. Our results are in accordance with those of Diacono et al. [18], who found savings of 25% of the amount of fertilizer with variable rate fertilization without reducing yield or grain quality.

Our data also suggest that where other limiting factors exist, applying high doses of N does not provide yield advantages and does not provide the best results in terms of efficiency. This does not agree with results of Guerrero et al. [17] who found that high N doses in low-yielding zones represent the best variable rate practice. In our case soil-based VRT was coherent with the principles of sustainability (less fertilizer, good qualitative-quantitative yield response and efficient use of the resources). The economic profitability of differential management has been questioned [35–37] with the argumenta that returns from PF technologies does not cover their costs or does not significantly change profitability [38], particularly when yield response to N does not vary strongly within the field [39]. However, in these analyses the positive effect of efficient resource use on environmental aspects and on down-side risk mitigation, are not taken into account [38]. Further research needs to consider such and other economic aspects: despite a general reduction of production costs and increase in gross margin, in accordance with Fabiani et al. [40] the high cost in machinery needed is a constraint to adopt VRT considering the generally small sizes of Italian farms. Also, the complexity of decisions from sensor data to agronomic management is one of the main challenges for the application of digitalization in agriculture. Simple approaches like the one presented in our study based on soil information versus more complex multi-data approaches may help simplify decisions and reduce costs.

## 5. Conclusions

In our experiment coupling geophysical mapping and traditional nitrogen balance was able to provide a quick basis for precision farming and VRT for N fertilization of wheat. Satisfactory production levels were reached by adapting the fertilization inputs to soil spatial variability. Yield and quality of UA was the same respect to VRT, but at the cost of more fertilizer, therefore less efficient in resources use efficiency. Also, in VRT all the N given with the fertilizer was taken up by the crop, while in the case of UA 22% of the N applied was not, and was instead left in the soil and this implies a higher environmental risk of soil-water pollution.

Results from this research indicate that coupling geophysical mapping and traditional nitrogen balance may provide a quick basis for precision farming and VRT for N fertilization of wheat.

## Supporting information

S1 Data Fig 4. (XLSX)

S2 Data Fig 5. (XLSX)

S3 Data Tables 3 and 4. (XLSX)

## References

[1] Guerrini L, Napoli M, Mancini M, Masella P, Cappelli A, Parenti A, et al. Wheat Grain Composition, Dough Rheology and Bread Quality as Affected by Nitrogen and Sulfur Fertilization and Seeding Density. Agronomy 2020; 10(2): 233. 10.3390/agronomy10020233

[2] Rathore VS, Nathawat NS, Bhardwaj S, Sasidharan RP, Yadav BM, Kumar M. Yield, water and nitrogen use efficiencies of sprinkler irrigated wheat grown under different irrigation and nitrogen levels in an arid region. Agric Water Manag. 2017; 187: 232–245. doi: 10.1016/j.agwat.2017.03.031

[3] Passioura JB. Environmental biology and crop improvement. Func Plant Biol. 2002; 29: 537–546. doi: 10.1071/FP02020 32689499

[4] Hawkesford MJ. Reducing the reliance on nitrogen fertilizer for wheat production. J Cereal Sci. 2014; 59: 276–283. doi: 10.1016/j.jcs.2013.12.001 24882935 PMC4026125

[5] Blandino M, Marinaccio F, Reyneri A. Effect of late-season nitrogen fertilization on grain yield and on flour rheological quality and stability in common wheat, under different production situations. Ital J Agron. 2016; 11(2): 107–113. 10.4081/ija.2016.745

[6] Hirel B, Le Gouis J, Ney, B, Gallais, A. The challenge of improving nitrogen use efficiency in crop plants: towards a more central role for genetic variability and quantitative genetics within integrated approaches. J Exp. 2007; Bot. 58: 2369–2387. doi: 10.1093/jxb/erm097 17556767

[7] Todorovic M., Caliandro A., Albrizio R. Irrigated agriculture and water use efficiency in Italy.In: Lamaddalena N. (ed.), Shatanawi M. (ed.), Todorovic M. (ed.), Bogliotti C. (ed.), Albrizio R. (ed.). Water use efficiency and water productivity: WASAMED project. Bari: CIHEAM, 2007. p. 101–136. (Options Méditerranéennes: Série B. Etudes et Recherches; n. 57). 4. WASAMED (WAter SAving in MEDiterranean agriculture) Workshop, 2005/09/30-2005/10/04, Amman (Jordan). http://om.ciheam.org/om/pdf/b57/00800782.pdf

[8] Mateo-Marín N, Quílez D, Isla R. Utility of stabilized nitrogen fertilizers to reduce nitrate leaching under optimal management practices. J Soil Sci Plant Nutr. 2020; 183(5): 567–578. doi: 10.1002/jpln.201900561

[9] Basso B, Cammarano D, Fiorentino C, Ritchie JT. Wheat yield response to spatially variable nitrogen fertilizer in mediterranean environment. Eur J Agron. 2013; 51: 65–70. doi: 10.1016/j.eja.2013.06.007

[10] Elmetwalli AH, Tyler AN. Estimation of maize properties and differentiating moisture and nitrogen deficiency stress via ground–based remotely sensed data. Agric Water Manag. 2020; vol. 242. doi: 10.1016/j.agwat.2020.106413

[11] Fabbri C, Mancini M, Dalla Marta A, Orlandini S, Napoli M. Integrating satellite data with a nitrogen nutrition curve for precision top-dress fertilization of durum wheat. Eur J Agron. 2020; Vol.120. doi: 10.1016/j.eja.2020.126148

[12] Kitchen NR, Sudduth KA, Drummond ST, Scharf PC, Palm HL, Roberts DF, et al. Ground-based canopy reflectance sensing for variable-rate nitrogen corn fertilization. Agron J. 2010; 102: 71–84. 10.2134/agronj2009.0114

[13] MIPAAF. “Linee guida per lo sviluppo dell’agricoltura di precisione in Italia”, Ministero delle politiche agricole alimentari e forestali, Gruppo di lavoro per lo sviluppo dell’Agricoltura di Precisione, 2015.

[14] Balafoutis A, Beck B, Fountas S, Vangeyte J, Wal T, Soto I, et al. Precision agriculture technologies positively contributing to GHG emissions mitigation, farm productivity and economics. Sustainability 2017; 9 (8): 1339. 10.3390/su9081339

[15] Rogovska N, Laird DA, Chiou CP, Bond LJ. Development of field mobile soil nitrate sensor technology to facilitate precision fertilizer management. Precis Agric. 2019; 20: 40–55. 10.1007/s11119-018-9579-0

[16] Rutting T, Aronsson H, Delin S. Efficient use of nitrogen in agriculture. Nutr Cycl Agroecosyst. 2018; 110: 1–5. 10.1007/s10705-017-9900-8

[17] Guerrero A, De Neve S, Mouazen AM. Data fusion approach for map-based variable-rate nitrogen fertilization in barley and wheat. Soil Tillage Res. 2021; Vol. 205. doi: 10.1016/j.still.2020.104754 33390631 PMC7729824

[18] Diacono M, Rubino P, Montemurro F. Precision nitrogen management of wheat. A review. Agron Sustain Dev. 2013; 33(1): 219–241. doi: 10.1007/s13593-012-0111-z

[19] Rossi R, Amato M, Bitella G, Bochicchio R. Electrical resistivity tomography to delineate greenhouse soilv ariability. Int Agrophys. 2013; 27: 211–218. 10.2478/v10247-012-0087-6

[20] Basso B, Cammarano D, Chen D, Cafiero G, Amato M, Bitella G, et al. Landscape Position and Precipitation Effects on Spatial Variability of Wheat Yield and Grain Protein in Southern Italy. J Agron CropSci. 2009; 195: 301–312. 10.1111/j.1439-037X.2008.00351.x

[21] Bitella G, Rossi R, Loperte A, Satriani A, Lapenna V, Perniola M, et al. Geophysical Techniques for Plant, Soil, and Root Research Related to Sustainability. In: The Sustainability of Agro-Food and Natural Resource Systems in the Mediterranean Basin, Vastola A. (ed.), Springer-Verlag Berlin and Heidelberg, 978-3-319-16356-7 (Print) 978-3-319-16357-4 (Online), 2015; 353–372. 10.1007/978-3-319-16357-4_23

[22] Morari F, Castrignanò A, Pagliarin C. Application of multivariate geostatistics in delineating management zones within agravelly vineyard using geo-electrical sensors. Comput Electr Agric. 2009; 68: 97–107. 10.1016/j.compag.2009.05.003

[23] Pollice A, Jona Lasinio G, Rossi R, Amato M, Kneib T, Lang S. Bayesian Measurement Error Correction in Structured Additive Distributional Regression with an Application to the Analysis of Sensor Data on Soil-Plant Variability. Stoch Environ Res Risk Assess. 2019; 33(3): 747–763. 10.1007/s00477-019-01667-1

[24] Rossi R, Pollice A, Bitella G, Labella R, Bochicchio R. Amato M (2018). Modelling the non-linear relationship between soil resistivity and alfalfa NDVI: a basis for management zone delineation, J. Appl. Geophys, 159: 146–156.

[25] Mori M, Di Mola I. Guida alla concimazione: metodi, procedure e strumenti per un servizio di consulenza. Imago Editrices.r.l., Rimini, Italy, 2012.

[26] Page AL, Miller RH, Keeney DR. Methods of Soil Analysis. In Chemical and Microbiological Properties, 2nd Edition; Agronomy Society of America, Madison, WI, 1982; Part 2.

[27] Soil Survey Staff, Keys to Soil Taxonomy, 2014. USDA-Natural Resources Conservation Service, 12th ed. Washington, DC.

[28] Moll RH, Kamprath EJ, Jackson WA. Analysis and interpretation of factors which contribute to efficiency of nitrogen utilization. Agron J. 1982; 74: 562–564. 10.2134/agronj1982.00021962007400030037x

[29] R Core Team. A Language and Environment for Statistical Computing; R Foundation for Statistical Computing: Vienna, Austria, 2018. Available online: https://www.R-project.org.

[30] Song X, Wang J, Huang W, Liu L, Yan G, Pu R. The delineation of agricultural management zones with high resolution remotely sensed data. Precis Agric. 2009; 10: 471–487. doi: 10.1007/s11119-009-9108-2

[31] De Mastro F, Cocozza C, Traversa A, Savy D, Abdelrahman HM, Brunetti G. Influence of crop rotation, tillage and fertilization on chemical and spectroscopic characteristics of humic acids. PLoS ONE 2019 a; 14 (6): e0219099. 10.1371/journal.pone.0219099 31247049 PMC6597113

[32] De Mastro F, Brunetti G, Traversa A, Cocozza C. Effect of crop rotation, fertilisation and tillage on main soil properties and its water extractable organic matter. Soil Res. 2019 b; 57: 365–373. 10.1071/SR18297

[33] Benincasa P, Antognelli S, Brunetti L, Fabbri CA, Natale A, Sartoretti V, et al. Reliability of ndvi derived by high resolution satellite and uav compared to in-field methods for the evaluation of early crop n status and grain yield in wheat. Exp Agric 2018; 54(4): 604–622. doi: 10.1017/S0014479717000278

[34] Diacono M, Troccoli A, Girone G, Castrignanò A. Field-scale variability and homogeneous zone delineation for some qualitative parameters of durum wheat semolina in Mediterranean environment. World J Agr Sci. 2011; 7 (3): 286–290. doi: 10.1007/s13593-012-0111-z

[35] OECD. Farm Management Practices to Foster Green Growth, OECD Green Growth Studies, OECD Publishing, Paris, 2016. 10.1787/9789264238657-en

[36] Gandorfer M, Meyer-Aurich A. Economic potential of site-specific fertiliser application and harvest management. In Precision Agriculture: Technology and Economic Perspectives, Cham: Springer International Publishing; Pedersen S. M. & Lind K. M. (Eds.); 2017; 79–92. 10.1007/978-3-319-68715-5_3

[37] Karatay YN, Meyer-Aurich A. Profitability and downside risk implications of site-specific nitrogen management with respect to wheat grain quality. Precis Agric.2020; 21(2): 449–472. doi: 10.1007/s11119-019-09677-3

[38] Yost MA, Kitchen NR, Sudduth KA, Massey RE, Sadler EJ, Drummond ST, et al. A long-term precision agriculture system sustains grain profitability. Precis Agric. 2019; 20: 1177–1198. 10.1007/s11119-019-09649-7

[39] Liu Y, Swinton SM, Miller NR. Is site-specific yield response consistent over time? Does it pay? Am J Agric Econ. 2006; 88(2): 471–483. 10.1111/j.1467-8276.2006.00872.x

[40] Fabiani S, Vanino S, Napoli R, Zajícek A, Duffková R, Evangelou E, et al. Assessment of the economic and environmental sustainability of Variable Rate Technology (VRT) application in different wheat intensive European agricultural areas. A Water energy food nexus approach. Environ Sci Policy 2020; 114: 366–376. https://doi.org/2-s2.0–85090883104

